# Dynamical Modeling and Analysis of Viscoelastic Properties of Single Cells

**DOI:** 10.3390/mi8060171

**Published:** 2017-06-01

**Authors:** Bo Wang, Wenxue Wang, Yuechao Wang, Bin Liu, Lianqing Liu

**Affiliations:** 1 State Key Laboratory of Robotics, Shenyang Institute of Automation, Chinese Academy of Sciences, Shenyang 110016, China; wangbo@sia.cn (B.W.); ycwang@sia.cn (Y.W.); liubin@sia.cn (B.L.); 2 University of Chinese Academy of Sciences, Beijing 100049, China

**Keywords:** dynamical modeling, mechanical properties, principal component analysis, atomic force microscopy, viscoelasticity

## Abstract

A single cell can be regarded as a complex network that contains thousands of overlapping signaling pathways. The traditional methods for describing the dynamics of this network are extremely complicated. The mechanical properties of a cell reflect the cytoskeletal structure and composition and are closely related to the cellular biological functions and physiological activities. Therefore, modeling the mechanical properties of single cells provides the basis for analyzing and controlling the cellular state. In this study, we developed a dynamical model with cellular viscoelasticity properties as the system parameters to describe the stress-relaxation phenomenon of a single cell indented by an atomic force microscope (AFM). The system order and parameters were identified and analyzed. Our results demonstrated that the parameters identified using this model represent the cellular mechanical elasticity and viscosity and can be used to classify cell types.

## 1. Introduction

Cells are the basic component units of organisms and contain important and abundant biological information. The complete genetic information of humans can be obtained from a single cell [1]. Furthermore, a cell is a complex network that contains thousands of overlapping signaling pathways. Traditional methods for describing the dynamics of this network are extremely complicated. Zhang et al. described the local dynamic behavior of a cellular signal network using 23 equations and 82 parameters [2]. Otte et al. used a 118-equation model with 177 parameters to describe the dynamics of ion channels [3]. Moreover, it is difficult to measure variations in the chemical components of the pathways of the network in a living cell. Therefore, it is difficult to analyze the global properties of cells via their underlying mechanisms, let alone to determine how to control them.

The mechanical properties of a cell reflect the structure and composition of the cytoskeleton and play a significant role in the regulation of cell physiology; therefore, they are closely related to the cell behavior, such as that in cell growth, division, differentiation, proliferation, migration, and adhesion. Recently, some studies showed that variation in the mechanical properties of cells is associated with the emergence and development of human disease [4]. Many diseases—e.g., cancer—can drastically affect the mechanical properties at the cellular level [4,5,6]. The development of nanotechnologies, including the atomic force microscope (AFM), magnetic and optical tweezers [7], and micropipettes [8], has enabled the measurement of the mechanical properties of single living cells. Thus, cellular mechanical information can be utilized as a label-free biomarker for cell recognition [9], early diagnosis of disease, and drug efficacy evaluation [10]. Additionally, studying the mechanical properties of single cells may provide a potential method for the detection of abnormal cells, early diagnosis of serious disease, and drug screening. Therefore, it is important to measure and quantitatively describe the mechanical properties of a single cell using a mathematical model.

The AFM has been widely used to measure and investigate the mechanical properties of living cells. To determine cellular mechanical properties using an AFM, the AFM indentation process, generally comprising three interaction phases—approach, stress-relaxation, and retraction—between the AFM tip and sample cells, is usually implemented to obtain force-indentation curves, from which the mechanical properties can be calculated using various theories and models. The Hertz model [11] is a widely-used model to describe the relationship between the force and indentation depth—i.e., the deformation of cells—and has various mathematical expressions depending on the probe shape, including pyramid [12], cone [13], and sphere [14,15]. Therefore, the Young’s modulus of cells can be statistically calculated using the Hertz model for the approach phase of the force-indentation curves. However, the Hertz model has some issues that need to be addressed. For example, the Hertz model assumes that the measured materials are linearly elastic, isotropic, and lack adhesion and friction [16], which does not hold for cells. Due to the assumption of linear elasticity for cells, the Hertz model cannot explain the dynamic stress-relaxation behavior commonly observed in living cells [17]. A.H.W. Ngan et al. developed a rate-jump approach to evaluate the elastic modulus of viscoelastic materials, including polymethyl methacrylate and living cells, using AFM indentation by applying a sudden step change in the loading rate on the sample, in which the viscous response is ignored [18,19,20,21]. However, in this method, the elasticity of the sample materials is still assumed to be linear and, considering the high complexity of the viscoelastic properties of cells, the analytical solution of elasticity is not yet accurate enough to characterize the mechanics of living cells. Using a three-element standard solid model, J. R. Dutcher et al. extracted the elastic and viscous properties of the bacterial cell envelope separately from the time-dependent creep deformation curve, which resulted from a constant force [22]. Similarly, A. Yango et al. applied a linear standard solid model to calculate the elastic and viscous properties of soft materials from the creep response to the loading and unloading steps during the stress-relaxation phase of AFM indentation [23]. In these two methods, the elastic and viscous properties are decoupled from the indentation force curves with the standard linear solid model; however, both methods assume that the soft materials are a first-order system, which ignores the high orders of the complex viscoelasticity properties of living cells. Recently, Wei et al. developed a rectification approach using finite element simulation with the assumption that the cell material is viscoelastic and has acquired the viscosity and elasticity parameters that reflect the actual dynamical mechanics of cells [24]. However, this finite element simulation-based approach does not provide an analytical solution that describes the system dynamics of cells for further system analysis. 

In general, the more complicated a system is, the harder it is to model based on the underlying mechanisms. Furthermore, the mechanism-based models are extremely complicated with high order and nonlinearities, especially for organisms, making the model analysis difficult. However, a system can be dynamically modelled based on the system input and output without considering its activity mechanism. In this work, a cell was considered a dynamical system, and a linear dynamical system model with cellular viscoelastic properties as system parameters was established to describe the stress-relaxation phenomenon of cells under the indentation of the AFM cantilever. The system order was determined by system identification using the Hankel matrix method, and the system parameters—i.e., the viscoelastic properties of cells—were identified using the least squares method. The viscosity and elasticity parameters were then used for cell classification. In this work, the system order of cells was not pre-assumed and was instead determined using the system identification method and experimental data. In this model, the viscosity and elasticity properties of cells are decoupled, and each is represented by multiple parameters. 

## 2. Materials and Methods

In this section, the experimental process of obtaining the input and output curves is first described. The AFM indentation process consists of three interaction phases—approach, stress-relaxation, and retraction. Next, a general Maxwell model to describe the indentation process is given, and the process of modeling the mechanical properties of a single cell is described. After determining the general form of the cellular system model, we used the Hankel matrix method to determine the order of the general model and the least squares method to determine the parameter in the system model. Finally, we performed mechanical parameter extraction under the viscoelastic assumption to describe the mechanical properties of a single cell.

### 2.1. Cell Preparation

The cell lines used in this study were obtained from the Institute Pasteur of Shanghai, Chinese Academy of Sciences (Shanghai). MCF-7 cells (human breast cancer cell line), L-929 cells (mouse fibroblast cell line), Neuro-2a cells (Mus musculus brain neuroblastoma cell line), and HEK-293 cells (human embryonic kidney cell line) were cultured in RPMI-1640 (Thermo Scientific HyClone, Logan, UT, USA) containing 10% fetal bovine serum and 1% penicillin-streptomycin solution at 37 °C (5% CO_2_). These four types of cells were cultured in Petri dishes. The diameter of the Petri dishes we used was 60 mm, and the cell concentration was about 1.3 × 10^6^ cm^−2^. The cells were cultured for 24 h before experiments. The same experiments were performed with different batches of cells. All the AFM experiments in this study were performed in culture medium. The experiments were conducted at room temperature. In this study, twenty cells of each type were selected, and two complete indentation processes were implemented independently for each cell. Therefore, 160 force curves were recorded, and the system order identification and parameter estimation process was performed for all cells using the experimental data.

### 2.2. Indentation Process

**Figure 1:**
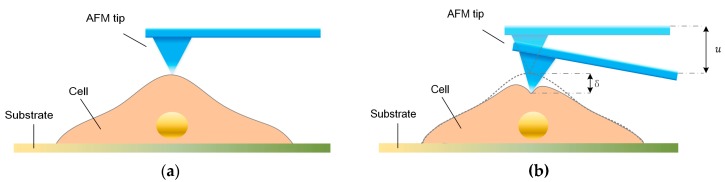
Illustration of the atomic force microscope (AFM) indentation experiment and the experimental curve from one entire indentation process. (**a**,**b**) Schematic diagram of indentation applied using an AFM probe tip on a single cell.

To determine the mechanical properties of cells using the stress-relaxation curve with an AFM, an indentation process was implemented, and the schematic of the AFM indentation is shown in Figure 1a,b. The entire process consists of three interaction phases—i.e., approach, stress-relaxation, and retraction. During the approaching phase, the AFM probe tip is pressed on the sample cell, causing a fast cell deformation and a rapidly increasing force on the cantilever. During the stress relaxation phase, the piezoelectric (PZT) actuator is kept at a constant depth, but the cell continues to deform and cause gradual decreases in the cantilever deflection. During the retraction phase, as the AFM probe tip is retracted, the cell recovers and the cell deformation rapidly decreases. Notably, the distance that the cell is indented is not equal to the distance u(t), that the PZT moves, and the difference between these two distances is the cantilever deflection. Furthermore, during the stress-relaxation phase, the deformation rate of cells becomes slower, and the cell gradually approaches constant deformation; therefore, the force between the tip and cell become constant. In this study, we used a Bioscope Catalyst AFM (Bruker, Billerica, MA, USA) and an inverted microscope (Nikon, Tokyo, Japan). The type of AFM probe used in this study was MLCT (Bruker), and the nominal spring constant of the cantilever used was 0.01 N/m. We used the thermal tune to calculate the spring constant of the cantilever. And the actual value of the spring constant was about 0.2960 N/m. The width of the cantilever was about 0.59–0.61 μm, and the aspect ratio of the AFM tip was about 3.78. In this study, we used the same cantilever to probe all the cells in the experiment. The movement speed of the AFM PZT in the indentation experiments was 4 μm/s during both the approach and retraction phases. The stress-relaxation time was 6 s. In the experiment, the maximum distance of cantilever displacement was 1 μm. As mentioned above, the indentation depth on the cells was not equal to the distance that the PZT moved. The indentation depth of each cell was about 0.6–0.7 μm. The thicknesses of the cells were about 4–7 μm. For all of the AFM experiments we performed in this study, we measured the stress-relaxation curves of the cells in the nuclear region.

### 2.3. Dynamical Modeling of the Viscoelastic Properties of a Single Cell

**Figure 2:**
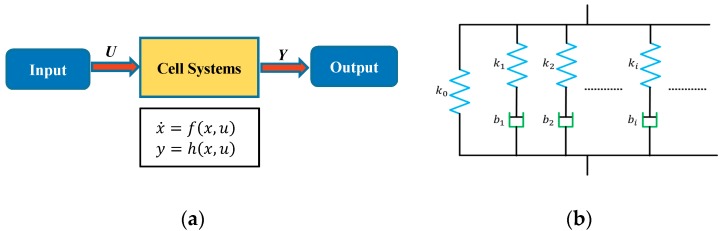
(**a**) Schematic diagram showing the system sciences point of view of cell dynamics; (**b**) An *n*th-order general Maxwell model of viscoelastic materials.

In this study, system sciences were used to model the dynamical mechanical behavior of a cell with its viscoelastic properties as the system parameters, based on the input (stimuli) and corresponding output (responses) instead of the interior structure of the cells. In this approach, as shown in Figure 2a, a single cell is considered as a general system and generates an output response under a certain input stimulus. The state variable x describes the internal dynamics of cell systems and constitutes the system output response y under the input stimuli u. During the AFM indentation process, as the AFM PZT moves down, the probe tip on the cantilever presses on the cell and causes the cell to deform. On the other hand, the indentation depth is less than the PZT movement distance, which leads to the deflection of cantilever. The cantilever deflection reflects the interaction force between the probe tip and the sample cell, and therefore, the interaction force can be measured from the cantilever deflection with the coefficients of the cantilever. Therefore, in this study, the AFM PZT *z*-position during the indentation is used as the input signal u(t), and the measured force is used as the output response y(t) of the cell system. In this study, the dynamic behaviors of a cell during the indentation process is modelled using a system approach without pre-assumption of the system order. The system order is determined with a system science approach. One of such approaches is the Hankel matrix method, in which a Hankel matrix is constructed from the impulse response series. The signals during the stress-relaxation phase can be regarded as the step response to the constant input (the PZT distance) to the system, and we can obtain the impulse response series by calculating the difference between every two adjacent points in the step response sequence. Therefore, for convenience, only the signals during the stress-relaxation phase are used for modeling and further analysis. The output force curve during the stress-relaxation phase showed the tendency for an exponential decay under a constant input for the PZT *z*-position. Therefore, a general Maxwell model for viscoelastic materials containing a spring and *n* parallel spring-damping paths, as shown in Figure 2b, was used to model the cell dynamics of the cell system. Mathematically, the cell system can then be written using a state-space equation as follows:

(1) $$\{\begin{array}{l} {\dot{x}}_{i}=-\frac{k_{i}}{b_{i}}x_{i}+\frac{k_{i}}{b_{i}}u\;,\;i=1,2,\ldots ,n\\ y=-\sum_{i=1}^{n}k_{i}x_{i}+\left(k_{0}+\sum_{i=1}^{n}k_{i}\right)u \end{array}$$

where u and y are the system input and output, respectively, the state variable $x_{i}$ represents the movement distance of the point between the spring and damper in the *i*th path, and $k_{i}$ and $b_{i}$ are the elastic and viscous parameters of the corresponding springs and dampers, respectively. The states $x_{i}$ were closely related to the cell deformation.

This model illustrates that the elasticity and viscosity of cells can be represented by the multiple parameters $k_{i}$ and $b_{i}$. Next, with the data collected in the indentation experiments, the order *n* of the cell system and parameters can be determined by system identification methods.

### 2.4. Order and Parameters Identification

The dynamical deformation behavior of a cell subjected to a constant indentation depth has been modelled by a linear dynamical model based on the structure of the general Maxwell model. In this section, the order and parameters of the cell system need to be determined from the input and output data. In this study, we used the Hankel matrix method to determine the order of the linear system. For linear systems, the Hankel matrix method is a classical approach for determining the order [25]. In this method, Hankel matrices are built from the impulse response sequence of the system, and the order of the system is actually the rank of the Hankel matrices. The criterion for identifying a system order using Hankel matrices is described in the following lemma.

**Lemma** **1.** *Let* {g(i)|i=1, 2,…,L} *be the impulse response sequence of a linear system. Hankel matrices can be constructed as follows:*

$$H\left(l,k\right)=\left[\begin{array}{ccc} \begin{array}{cc} g\left(k\right) & g\left(k+1\right)\\ g\left(k+1\right) & g\left(k+2\right) \end{array} & \cdots & \begin{array}{c} g\left(k+l-1\right)\\ g\left(k+l\right) \end{array}\\ \vdots & \ddots & \vdots \\ \begin{array}{cc} g\left(k+l-1\right) & g\left(k+l\right) \end{array} & \cdots & g\left(k+2l-1\right) \end{array}\right]$$

*where* l *determines the dimension of the Hankel matrix, and* k *is any integer between* 1 *and* L−2l+2*. The order of the system is equal to the rank* $n_{0}$ *of the Hankel matrices if*

(2) $$rank\left[H\left(l,k\right)\right]=n_{0},\;for\;all\;l\geq n_{0},\;\forall k$$

This criterion works perfectly for noise-free data. In general, the impulse response sequence includes noise, so the rank of the Hankel matrix may be not equal to $n_{0}$ exactly, even when l ≥ $n_{0}$. Therefore, an equivalent criterion using the average ratio of the determinant of Hankel matrix $D_{l}$ is used to evaluate the singularity of the Hankel matrices and the order of the dynamical systems, where

(3) $$D_{l}=\frac{\frac{1}{L-2l+2}\sum_{k=1}^{L-2l+2}\det[H(l,k)]}{\frac{1}{L-2l}\sum_{k=1}^{L-2l}\det[H(l,k)]}$$

in which l is the dimension of the Hankel matrices and is not equal to 1. The determinant $D_{l}$ grows as l increases if $l<n_{0}$ and then decays for $l>n_{0}$, and reaches a maximum at $l=n_{0}$. Therefore, the value of l at which $D_{l}$ reaches the maximum value can be considered to be the order of the system. In this study, we used $D_{l}$ to evaluate the order of the cell system.

In practice, the impulse response sequence of a dynamical system can be obtained by calculating the difference between every two adjacent points in the step response sequence of the system, i.e., g(i)=y(i+1)−y(i), i=1,2,…,L, where g(i) is the *i*th element in the impulse response sequence, and y(i) is the *i*th element in the step response sequence. In our study, the input stimulus is a constant indentation depth during the stress-relaxation phase of the indentation process. Therefore, the output of the cell system, the interaction force between the probe tip and the cell, is actually the step response. Hence, we can obtain the impulse response sequence from the recorded force curves and construct the Hankel matrices. 

Once the order of the dynamic system of a single cell is determined, the parameters of the system can be easily determined by the least squares method. In this method, the parameters are chosen to minimize the error between the system model output and experimental data, i.e.;

(4) $$\theta^{*}=arg\underset{\theta \in \Theta }{\min}\|y_{\theta }\left(t\right)-\bar{y}{\left(t\right)\|}_{2}$$

where $y_{\theta }\left(t\right)$ is the model output with the parameter $\theta =\left[k_{0},k_{1},b_{1},k_{2},b_{2}\right]$ in Equation (1), Θ is the corresponding parameter space of θ, $\bar{y}\left(t\right)$ is the actual output of a single cell measured by AFM in the experiments, and the parameter $\theta^{*}$ represents the system parameter minimizing the error. The system identification toolbox of MATLAB (R2016b, MathWorks, Natick, MA, USA) is used in this study to identify the system parameters using the least squares method.

Now we have introduced approaches for identifying the system order and estimating the system parameters of dynamical cell systems. Given the system order and parameters, the corresponding dynamic equations can describe the dynamical behavior during the stress-relaxation process and the mechanical properties of a single cell completely under the condition of viscoelastic assumptions.

## 3. Results and Discussion

**Figure 3:**
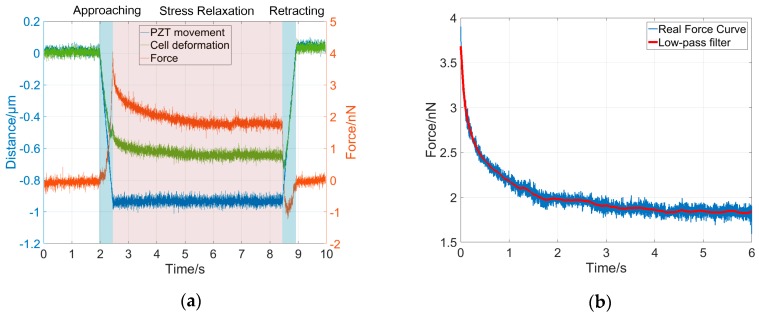
Illustration of the experimental curve from one entire indentation process. (**a**) The entire indentation process showing the changes in the *z*-position of piezoelectric (PZT) (blue), cell deformation (green), and interaction force (red) that the AFM measures during the three interaction phases; (**b**) Original force curve (blue) in the stress-relaxation phase and smoothed curve using a low-pass filter.

To validate the dynamical model for cell deformation behaviors under a constant indentation depth, we performed indentation experiments using four types of cells, namely MCF-7, HEK-293, L-929, and Neuro-2a cells, and the corresponding stress-relaxation force curves were collected for system order and parameter analysis. The cells responded with different dynamic behaviors at different stages of the indentation experiment, as shown in Figure 3a. In this study, each indentation experiment lasted about 8 s, including 1 s for the approaching phase, 6 s for the stress-relaxation phase, and 1 s for the retraction phase. During the approach phase, the AFM probe tip was pressed onto the sample cells and moved down along with the constant movement of the PZT (blue curve in Figure 3a), resulting in a fast cell deformation (green curve in Figure 3a) and a rapidly increasing force on the AFM cantilever (red curve in Figure 3a). During the stress-relaxation phase, the PZT was kept at a constant depth (*z*-position) which was equal to 1 μm, however, the cell continued to be further deformed due to its viscosity and therefore the cantilever deflection and the interaction force between the tip and cell gradually decreased. During the retraction phase, as the AFM probe tip was retracted, the cell recovered, and therefore the cell deformation and the interaction force on the cantilever rapidly decreased. The relaxation time of a cell during the stress-relaxation phase in the indentation process depends on its mechanical properties, which were characterized by the time constants, that is to say, by the ratio of the elasticity parameters to the viscosity parameters, as shown in Equation (5). Therefore, for different types of cells, the relaxation times are different. In general, the stress-relaxation time needs to last until the stress-relaxation curve remains steady after a decline. Darling et al. showed that the stress-relaxation curves of the chondrosarcoma cells remained steady after 20 s [17]. Moreover, the response time of cells is also related to the type of stimulus signal [16,26,27,28]. In our study, for each type of cell, the stress-relaxation curve remained steady after 4 s. The indentation depth was about 0.6–0.7 μm, and the maximum interaction force between the tip and the cell was less than 5 nN. In this study, only the force curves during the stress-relaxation phase were used to validate the dynamical model and to identify the system parameters. To reduce the noisy effect in the measurement, a low-pass filter with a 10-Hz cut-off frequency was used to smooth the force curves for further analysis, as shown in Figure 3b.

**Figure 4:**
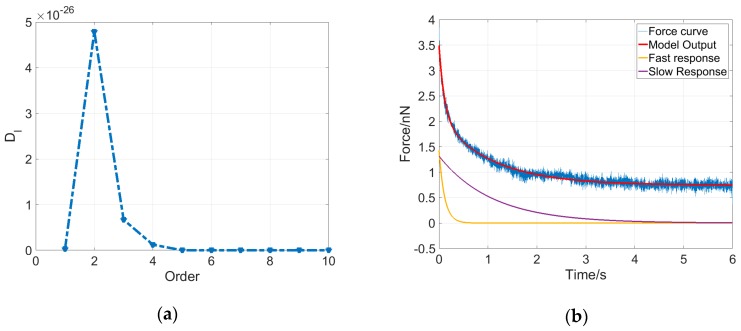
Determination of the system order and parameters of an MCF-7 cell. (**a**) $D_{l}$ series of an MCF-7 cell. As *l* increases, $D_{l}$ reaches the maximum at l = 2 and then decays to 0; (**b**) The model output of the cell system (red) of second order with estimated parameters fits the experimental force curve (blue) very well. The two exponential decay components represent the fast response (yellow) and slow response (purple) of the characteristics and the system dynamics of the MCF-7 cell.

As shown in Figure 4a, the determinant $D_{l}$ for an MCF-7 cell reaches the maximum at l=2; therefore, the dynamical system for this cell is determined to be a second-order system with five parameters, including three elasticity property parameters and two viscosity property parameters. Additionally, the output solution describing the cell dynamics can be written as follows:

(5) $$y\left(t\right)=k_{0}u\left(t\right)+u\left(t\right)k_{1}e^{-\frac{k_{1}}{b_{1}}t}+u\left(t\right)k_{2}e^{-\frac{k_{2}}{b_{2}}t}$$

Equation (5) indicates that the output of the system contains three components. If the system input u(t) is a constant, then the system output y(t) consists of a constant component and two exponential decay components. The five parameters were estimated using the least squares method, and then the system output could be obtained accordingly. As shown in Figure 4b, the system model output (red curve) of the deformation dynamics for the MCF-7 cell fits the experimental data (blue curve) very well, and two exponential decay components were plotted, indicating that the cell deformation dynamics is mainly dominated by a fast response at the very beginning of the stress-relaxation phase and then by a slow response for the remaining time.

**Figure 5:**
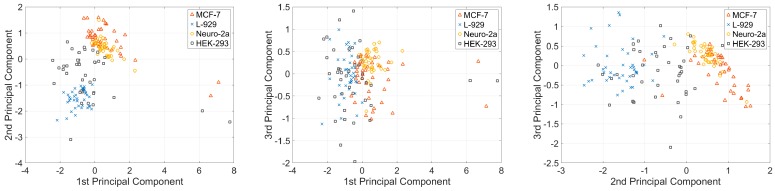
Principle components for four types of cells. These three plots show the first component vs. the second component, the first component vs. the third component, and the second component vs. the third component.

**Figure 6:**
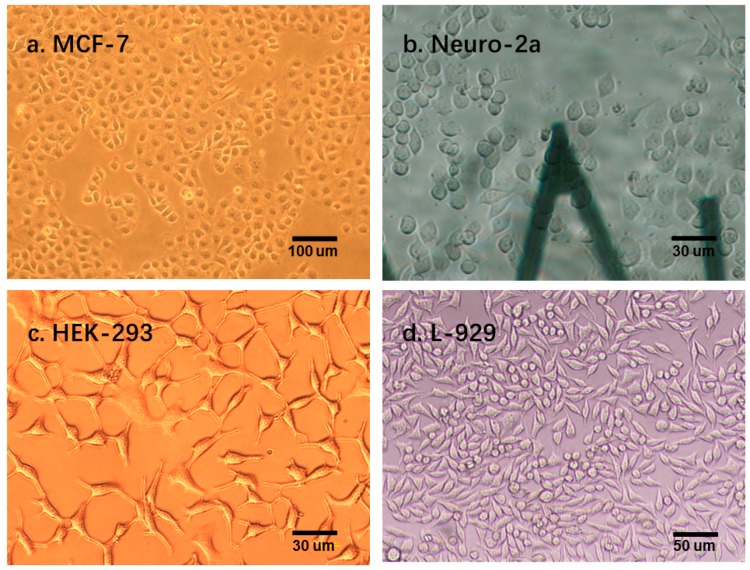
Morphology of four types of cells. (**a**) MCF-7 cells, (**b**) Neuro-2a cells, (**c**) HEK-293 cells and (**d**) L-929 cells. MCF-7 and Neuro-2a cells each showed less variance in morphology than HEK-293 and L-929 cells. The magnification is 10, 40, 40, and 20, respectively.

For all the cells, the dynamical system was determined to be second order. The system parameters were averaged for each type of cell, as indicated in Table 1. The vectors of these five parameters represent the elasticity $\left(k_{i}\right)$ and viscosity $\left(b_{i}\right)$ properties of a single cell; therefore, the vectors can be used to classify the cell types. Before classifying the cell types, we first conducted dimension reduction for the viscosity parameter vector to visualize the variations in the viscoelasticity parameters of cells in 2D spaces. In this study, the principal component analysis (PCA) method was used to reduce the dimension of the parameter vector. The main idea of PCA is to calculate the eigenvalue of the covariance matrix of the sample matrix. The first principle component has the largest eigenvalue, so it has the largest distinction degree. We calculated the sample matrix mentioned above, with the dimension of 160 × 5, and found that the eigenvalues of the first two principle components were larger than one, and the total contribution of the first three principle components was 96.35%. This finding indicates that the first three principle components include approximately 96.35% of the information of all five parameters (Table 2). The first three principle components of the four different types of cells are shown in Figure 5. Figure 5 shows that different types of cells present different clustering patterns; MCF-7 and Neuro-2a cells have more concentrated cluster patterns than the other two types of cells. This phenomenon may be attributed to the morphology variances of these four types of cells. As shown in Figure 6, MCF-7 and Neuro-2a cells each showed great morphology similarity, but HEK-293 and L-929 cells showed substantial variations in their shape and size. Multiple elasticity and viscosity parameters can be used to classify cell types.

**Figure 7:**
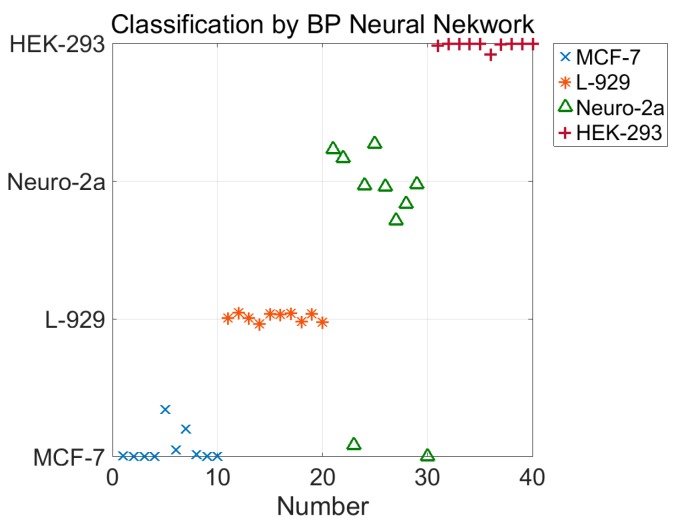
Classification result of the four types of cells by the backpropagation (BP) neural network. In the testing set, each type of cell has 10 test data. Among all 40 test data for the four cell types, only two points of Neuro-2a were misclassified. The success rate of the classification is 95%.

In this study, we used a backpropagation (BP) neural network to classify the four types of cells. The neural network has two hidden layers. The first hidden layer has 20 nodes and the second one has 40 nodes. The input of the neural network is the five parameters, $k_{0},k_{1},b_{1},k_{2},b_{2}$, and the output of the neural network is the type of cell, represented by 1 for MCF-7, 2 for L-929, 3 for Neuro-2a, and 4 for HEK-293. The training data set contains 120 five-parameter tuples, 30 for each type of cells. The test dataset contains 40 five-parameter tuples, 10 for each type of cell. The algorithm was implemented with MATLAB. The classification result using the BP neural network is shown in Figure 7. Among all 40 test data for the four cell types, only two points of Neuro-2a were misclassified. The success rate of the classification is 95%; this result further proves the validity of the model for characterizing the mechanical properties of cells.

## 4. Conclusions

In this study, using system science approaches, we presented a dynamical model based on the structure of a general Maxwell system to describe the cell deformation dynamics under conditions of a constant indentation depth during the stress-relaxation phase of the indentation process. The system order and parameters were identified using the Hankel matrix method and the least squares method, respectively. The proposed model was validated by AFM indentation experiments with different types of cells. Four types of cells with 20 cells of each type were evaluated to collect the stress-relaxation data to validate the proposed dynamical model; the dynamical system for all the cells used in this study was determined to be second order. Therefore, the system model contained three elasticity parameters and two viscosity parameters, which represent the elasticity and viscosity characteristics, respectively, of cell dynamics. In other words, with the proposed model, the nonlinear elasticity and viscosity properties of a single cell can be decoupled, and the nonlinearity characteristics of each property can be described by multiple parameters in a linear system. PCA of the five viscoelasticity parameters revealed different clustering patterns for the four types of cells, implying that the elasticity and viscosity parameters can be used to recognize the cell type. Future work will be conducted to investigate the effects of drugs on the elasticity and viscosity parameters of cells using the proposed model. 

## Figures and Tables

**Table 1:** Average parameters of cell four types.

Cellular Types	Elastic Parameters (N/m)			Viscosity Parameters (N·s/m)	
	$k_{0}$	$k_{1}$	$k_{2}$	$b_{1}$	$b_{2}$
MCF-7	2.984 ± 0.742	1.110 ± 0.372	1.385 ± 0.114	0.118 ± 0.306	3.108 ± 1.618
Neuro-2a	2.425 ± 0.589	1.519 ± 0.286	1.259 ± 0.121	0.242 ± 0.296	1.934 ± 0.891
Hek-293	1.099 ± 0.496	0.472 ± 0.203	0.609 ± 0.090	0.158 ± 0.244	1.956 ± 0.796
L-929	0.534 ± 0.096	0.195 ± 0.067	0.235 ± 0.009	0.0124 ± 0.049	0.374 ± 0.150

**Table 2:** Results of principle component analysis of cellular system parameters.

Principle Component	Eigenvalue	Difference Value	Contribution on Rate (%)	Total (%)
1st	3.3158	2.2298	66.3153	66.3153
2nd	1.0860	0.6685	21.7197	88.0351
3rd	0.4175	0.2631	8.3498	96.3849
4th	0.1544	0.1281	3.0884	99.4733
5th	0.0263	-	0.5267	100
